# Ordinal sentiment classification in cancer support forums: a controlled benchmark of machine learning and transformer models

**DOI:** 10.3389/fdgth.2026.1836536

**Published:** 2026-08-04

**Authors:** Zhongyan Wang, Yuchen Cao, Shuo Xu, Zhanyi Ding, Yijun Gao, Qinkai Mao, Tongsong Qi, Hailiang Wang

**Affiliations:** 1 Center for Data Science, New York University, New York, NY, United States; 2 Khoury College of Computer Sciences, Northeastern University, Boston, MA, United States; 3 Department of Computer Science and Engineering, University of California San Diego, La Jolla, CA, United States; 4 Krieger School of Arts and Sciences, Johns Hopkins University, Baltimore, MD, United States; 5 Weissman School of Arts and Sciences, Baruch College, City University of New York, New York, NY, United States; 6 Charles V. Schaefer, Jr. School of Engineering and Science, Stevens Institute of Technology, Hoboken, NJ, United States; 7 College of Computing, Georgia Institute of Technology, Atlanta, GA, United States

**Keywords:** ALBERT, cancer support forums, caregivers, natural language processing, ordinal sentiment, vulnerability

## Abstract

**Introduction:** Online cancer support forums contain naturalistic accounts of fear, uncertainty, coping, and caregiver strain. Such text may contribute to digital phenotyping as one component of longitudinal, human-supervised monitoring, but the operational link between psychosocial distress and sentiment labels requires explicit evaluation.

**Methods:** We benchmarked representative classical, recurrent, and transformer-based models for four-class ordinal sentiment classification using the *Mental Health Insights—Vulnerable Cancer Patients* dataset (*N* = 10,392). Models were evaluated under a single validation-guided 60/20/20 holdout split using weighted F1, macro one-vs-rest AUC, class-specific performance, and paired comparisons.

**Results:** Transformer models achieved the strongest overall performance. ALBERT produced the highest weighted F1 and macro AUC (0.7667 and 0.931, respectively), while BioBERT was closely comparable (weighted F1 = 0.7613; macro AUC = 0.917) and showed slightly higher recall for the “very negative” class (0.8019 vs. 0.7736). Error analysis showed that transformer errors concentrated around ordinal decision boundaries, while residual positive-class errors remained operationally important for supportive workflows.

**Discussion:** These split-specific findings support transformer fine-tuning as a decision-support component for vulnerability-oriented monitoring, while emphasizing calibration, transparent error review, and human oversight rather than autonomous clinical assessment.

## Introduction

Cancer patients and their informal caregivers often experience substantial psychosocial burden throughout diagnosis, treatment, and survivorship. Alongside clinical demands, many individuals face uncertainty, fear of recurrence, and disruptions to daily functioning, while caregivers may assume intensive coordination and emotional labor that can compound stress within the patient–caregiver dyad (1–3). In parallel with clinical care, an increasing share of coping, information seeking, and emotional disclosure occurs in online health communities, where patients and caregivers describe experiences in their own words and receive peer support outside formal appointments (4, 5). These digital traces can complement clinic-based assessment when interpreted cautiously as part of digital phenotyping: the use of passively or actively generated digital data to characterize behavior, affect, or functioning over time (6, 7).

In this study, we treat ordinal sentiment classification as one text-derived component of vulnerability-oriented digital phenotyping rather than as a stand-alone clinical assessment. The practical objective is to evaluate whether models can help summarize forum affect, prioritize posts for moderator or support-staff review, and flag potentially high-distress narratives for human-in-the-loop triage. This framing is intentionally narrower than diagnosis or automated intervention. It asks whether a controlled benchmark can inform model selection for downstream workflows in which predictions are interpreted by humans alongside context, platform policy, and supportive-care capacity.

Oncology-related forum language poses several challenges for this task. Sentiment cues are frequently context dependent, mixed within a single post (e.g., gratitude alongside fear), and expressed using medical terminology whose polarity may differ from everyday usage. Posts may also reflect multifactorial burden, including cognitive and symptom-related concerns, that can co-occur with emotional distress and blur boundaries between sentiment categories (8). Moreover, vulnerability-relevant affect is naturally ordinal: distinguishing acute distress from moderate negativity is often more consequential than merely separating negative from non-negative content, and evaluation should therefore consider not only accuracy but also the severity of misclassifications on an ordered scale (9). While confusing adjacent categories may result in manageable differences in review priority, distance-based errors, such as interpreting acute distress as neutral or positive, represent higher-risk failures.

### Related work

Prior work has used social media and online community data to study public health, mental health, and affective states at scale (10–12). Reviews of mental-health NLP emphasize both the promise of non-clinical text for early signal detection and the need for careful framing because labels, platforms, and populations differ substantially across studies (9, 13). In cancer and survivorship contexts, online support forums provide an especially relevant setting because patient and caregiver posts often combine informational needs, emotional disclosure, treatment uncertainty, and peer support (1, 3, 4). These properties make the text valuable for monitoring psychosocial burden, but they also increase annotation ambiguity and potential bias.

Machine learning approaches for affect and mental-health classification range from sparse lexical baselines to deep contextual encoders. Classical TF–IDF models offer computational efficiency and coefficient-level interpretability, while recurrent neural networks introduce sequence-sensitive representations (14, 15). Transformer architectures use self-attention to model contextual relationships and have demonstrated strong performance across NLP tasks (16). ALBERT improves parameter efficiency through cross-layer parameter sharing and factorized embeddings, making it attractive when labeled datasets are limited (17). BioBERT extends this paradigm through biomedical-domain pretraining, which may help with medically grounded expressions but may not fully capture the mixed affective and peer-support language typical of cancer forums (18). Recent benchmarking work in mental-health NLP further suggests that model choice is sensitive to preprocessing, class imbalance, and evaluation protocol, reinforcing the need for controlled comparisons rather than assuming that the most domain-specific or complex model is automatically preferable (19, 20).

Motivated by this literature, we benchmark representative classical ML and DL pipelines for four-class ordinal sentiment classification (very negative, negative, neutral, positive) in a cancer patient and caregiver vulnerability forum dataset. Rather than proposing a new architecture, our goal is to provide a controlled comparison that informs model selection for vulnerability-oriented monitoring in cancer support communities. The study contributes: (i) a comparison of linear, ensemble, recurrent, and transformer-based models for ordinal sentiment classification in cancer support discourse; (ii) evaluation with uncertainty estimation and paired hypothesis testing on held-out test predictions; and (iii) interpretation of per-class behavior and ordinal error patterns in terms of supportively salient distinctions, clarifying where transformer fine-tuning provides observed gains and where residual decision-boundary challenges remain.

## Methods

This study evaluated a suite of classical machine learning (ML) and deep learning (DL) architectures for four-class sentiment classification within a cancer support forum context. The implemented pipeline comprised: (i) automated text standardization and feature engineering, (ii) systematic model training utilizing grid and random search strategies, and (iii) uncertainty-aware statistical evaluation including bootstrapped interval estimation and paired hypothesis testing. To reduce optimistic bias, model selection was guided exclusively by performance on a held-out validation set, and final metrics were reported on an independent test set.

### Computational environment

All experiments were executed within a Python-based environment, using the core scientific stack of NumPy and Pandas for data manipulation. Classical ML workflows, including dataset partitioning, TF–IDF vectorization, and model fitting, were implemented via the scikit-learn framework (21). Gradient-boosted decision trees were developed using the LightGBM library (22). Deep learning models were built using PyTorch, with transformer architectures implemented through the Hugging Face transformers library, using pretrained checkpoints including ALBERT (17) (albert-base-v2) and BioBERT (18) (dmis-lab/biobert-base-cased-v1.2). Text preprocessing relied on the re module for regular expression-based cleaning and the Natural Language Toolkit (NLTK) for linguistic resources (23). Computational progress was monitored via tqdm, and hardware acceleration was utilized via CUDA for neural network training. GRU modeling followed the standard gated recurrent unit formulation (15).

### Dataset and label definition

We analyzed the *Mental Health Insights: Vulnerable Cancer Survivors & Caregivers* dataset (24) (also mirrored on Kaggle). The corpus consists of user-generated forum posts paired with sentiment annotations from the source dataset; the present study did not conduct a new annotation campaign. In the original release, two dataset annotators assigned each post an ordinal emotion score on a four-point scale from −2 to +1, where −2 and −1 indicate grief/suffering, 0 indicates neutral/no emotion, and +1 indicates positive emotions such as relief or accomplishment (24). The source dataset record identifies the annotator structure but does not report inter-annotator agreement statistics such as Cohen’s κ or Krippendorff’s α; therefore, the reliability ceiling imposed by label ambiguity cannot be quantified from the released materials. We mapped these scores to a four-class sentiment schema (very negative, negative, neutral, positive), yielding a final analytic sample of 10,392 post-level instances.

The unit of analysis was the individual post. Because the analytic file used for modeling did not contain stable user identifiers suitable for grouped splitting, the train/validation/test split should be interpreted as a held-out-post evaluation rather than a held-out-user evaluation. If multiple posts originated from the same forum participant, within-user dependence could remain; we therefore treat user-level generalization as an external-validity limitation rather than a demonstrated property of the present benchmark. Given the ordinal nature of sentiment intensity, labels were additionally mapped to an ordered integer scale: very negative = 0, negative = 1, neutral = 2, and positive = 3. This ordinal structure implies that misclassifications between adjacent categories (e.g., 0 and 1) are less severe than those between distant categories (e.g., 0 and 3), a distinction explicitly incorporated into our error analysis. Prior to model development, the dataset was shuffled with random_state =1234 to mitigate potential ordering artifacts.

### Text processing and feature construction

#### Text normalization

All models used the same cleaned post text as the input corpus before model-specific representation. The core difference was therefore representation rather than preprocessing: classical machine-learning models converted the cleaned text into sparse TF–IDF unigram/bigram vectors, whereas GRU, ALBERT, and BioBERT converted the cleaned text into token sequences for learned word embeddings or pretrained transformer tokenization. Thus, comparability came from shared labels, data split, validation-selection rule, and held-out test metrics, while each model family used the representation appropriate to its architecture.

#### Train/validation/test split

The data were partitioned using a two-stage holdout strategy to reduce leakage risk from hyperparameter selection and to provide a held-out final evaluation. Initially, 20% of the data was reserved for final testing. The remaining 80% was divided into 75% training and 25% validation subsets, resulting in a final 60/20/20 train/validation/test split. Both split operations were executed using random_state = 42. The validation set was used for hyperparameter selection, while the test set was reserved for final reporting.

#### Feature engineering

Classical ML models used TF–IDF vectorization with up to 10,000 features, including unigrams and bigrams (ngram_range = (1,2)). For deep learning, ALBERT used the AlbertTokenizer for albert-base-v2, BioBERT used the Hugging Face AutoTokenizer for dmis-lab/biobert-base-cased-v1.2, and both transformer inputs were padded or truncated to a maximum sequence length of 200 tokens. The GRU model used a task-specific vocabulary of the top 10,000 tokens, with sequences padded or truncated to length 200. Fixed sequence lengths and padding are standard requirements for minibatch training in neural text models, while TF–IDF dimensionality limits are standard controls for sparse lexical feature spaces (14, 16). These settings do not make the internal representations identical across model families; comparability instead comes from using the same labels, data split, validation-selection rule, and held-out test metrics.

### Model development and hyperparameter optimization

We evaluated models spanning linear, ensemble, and neural architectures. To account for class imbalance, training utilized class-weighted objective functions or balanced weighting strategies. We did not apply oversampling or text augmentation, because the primary aim was a controlled same-split benchmark across model families using the original released text. Model selection was performed using the validation weighted F1-score, which emphasizes performance across classes in proportion to their prevalence. Because a prevalence-weighted metric can obscure minority-class failures, weighted F1 was not interpreted alone: final reporting also included macro AUC, class-specific precision/recall/F1, support counts, and confusion-matrix-based error analysis. The specific hyperparameter search spaces for all evaluated models are consolidated in Table 1.

**Table 1:** Hyperparameter search spaces for ML and DL models.

Model	Hyperparameter	Search space
Grid Search (manual enumeration on validation set)		
Logistic Regression	Regularization (C)	{0.1,1,10}
	Solver	{lbfgs, saga}
	Class Weight	{balanced, None}
	Penalty	{l2} (fixed)
	Multiclass	{multinomial} (fixed)
Random Forest	Estimators (n_estimators)	{50,100,200}
	Max Depth	{None, 10, 20}
	Min Samples Split	{2,5,10}
	Min Samples Leaf	{1,2,4}
	Class Weight	{balanced, None}
LightGBM	Estimators (n_estimators)	{100,200}
	Learning Rate	{0.01,0.1,0.2}
	Number of Leaves (num_leaves)	{31,63}
	Max Depth	{None, 10}
	Class Weight	{balanced, None}
Random Search (n=10 samples)		
GRU	Embedding Dimension	uniform in [150,250]
	Hidden Dimension	uniform in [256,768]
	Learning Rate	log-uniform in $\left[10^{-4},10^{-3}\right]$
	Epochs	{5,6,7,8,9}
ALBERT	Learning Rate	log-uniform in $\left[10^{-5},10^{-4}\right]$
	Epochs	{3,4,5}
	Dropout	uniform in [0.0,0.25]
BioBERT	Learning Rate	log-uniform in $\left[10^{-5},10^{-4}\right]$
	Epochs	{3,4,5}
	Dropout	uniform in [0.0,0.25]

#### Logistic regression

Multinomial logistic regression was trained as an interpretable linear baseline on TF–IDF features using an l2 penalty (25). This model provides a strong, transparent reference point in high-dimensional sparse feature spaces, where coefficients offer direct insight into the direction and magnitude of association between lexical features and each sentiment class.

Hyperparameters were optimized via grid search, selecting the configuration that maximized validation weighted F1-score. The search space included the regularization strength C {0.1,1,10}, solver {lbfgs,saga}, and class weighting {balanced,None} (Table 1). The selected configuration thus reflects the best trade-off between shrinkage (generalization control), numerical optimization behavior (solver), and imbalance handling (class weights) under the study’s primary selection criterion.

#### Random forest

Random Forest was implemented as a tree-based bagging ensemble to capture non-linear feature interactions while reducing variance through bootstrap aggregation (26). This approach is well-suited to sparse TF–IDF representations because it can model higher-order interactions among features without requiring explicit feature engineering, while the ensemble structure improves robustness relative to single-tree models.

Hyperparameters were optimized via grid search with validation weighted F1-score as the selection criterion. The search space included the number of trees (n_estimators {50,100,200}), tree complexity (max_depth {None,10,20}), and split regularization (min_samples_split {2,5,10}; min_samples_leaf {1,2,4}), as well as class weighting {balanced,None} (Table 1). This tuning explicitly controls the bias–variance trade-off and mitigates overfitting by constraining tree growth and enforcing minimum sample requirements for splits and leaves.

#### LightGBM

LightGBM was utilized as a high-performance gradient boosting framework for high-dimensional TF–IDF features, employing a leaf-wise growth strategy and histogram-based feature binning for computational efficiency and strong predictive capacity (22). As a boosting method, LightGBM sequentially refines decision boundaries by fitting new trees to the residual errors of prior trees, enabling it to capture complex non-linear patterns that may be missed by linear models.

Hyperparameters were optimized via grid search with validation weighted F1-score used for model selection. The search space included the number of boosting iterations (n_estimators {100,200}), learning rate {0.01,0.1,0.2}, and tree-structure capacity (num_leaves {31,63}), alongside class weighting {balanced,None} (Table 1). These parameters jointly determine effective model capacity: n_estimators and learning rate control the strength and stability of boosting updates, while num_leaves governs the granularity of learned partitions.

#### GRU

A custom GRU architecture was developed to model sequential dependencies in token sequences with lower computational overhead than LSTM while maintaining the ability to capture long-range dependencies (15). The GRU model consisted of a randomly initialized task-specific embedding layer, a single GRU layer, and a fully connected output layer with 0.3 dropout, enabling the model to learn distributed representations while controlling overfitting through dropout regularization. It did not use pretrained word embeddings such as GloVe or fastText; accordingly, it should be interpreted as a lightweight randomly initialized recurrent baseline rather than the strongest possible representative of the recurrent architecture family.

GRU hyperparameters were optimized via 10 iterations of random search (Table 1), with the final configuration selected using validation weighted F1-score. The search space included embedding dimension [150,250], hidden dimension [256,768], learning rate $\left[10^{-4},10^{-3}\right]$ (log-uniform), and epochs {5,6,7,8,9}, while dropout was fixed at 0.3. This tuning explores representational capacity (embedding/hidden dimensions) and optimization dynamics (learning rate/epochs) while holding architectural regularization constant to isolate the effect of capacity and training schedule on validation performance.

#### ALBERT

We fine-tuned the pretrained albert-base-v2 transformer model for four-class sentiment classification (17). ALBERT leverages parameter sharing and factorized embedding parameterization to reduce memory footprint while retaining strong contextual representation learning, making it well-suited for modeling nuanced sentiment cues in forum narratives where meaning is often distributed across multi-token contexts.

ALBERT fine-tuning was optimized via 10 iterations of random search as specified in Table 1. The search space included learning rate $\left[10^{-5},10^{-4}\right]$ (log-uniform), dropout [0.0,0.25], and training epochs {3,4,5}. The final configuration was selected by validation weighted F1-score, balancing optimization stability (learning rate), regularization against overfitting (dropout), and training duration (epochs).

#### BioBERT

To assess the impact of domain-specific pretraining, we additionally fine-tuned BioBERT, a biomedical-domain transformer pretrained on large-scale biomedical corpora, for four-class sentiment classification. BioBERT is expected to better represent oncology- and symptom-related terminology frequently present in cancer support forum narratives, which may indirectly improve sentiment discrimination in medically grounded expressions.

BioBERT fine-tuning was optimized via 10 iterations of random search (Table 1). The search space included learning rate $\left[10^{-5},10^{-4}\right]$ (log-uniform), dropout [0.0,0.25], and training epochs {3,4,5}. To address class imbalance during training, we used a class-weighted cross-entropy objective with weights computed from the training labels using a balanced scheme. The final BioBERT configuration was selected by validation weighted F1-score.

### Model evaluation and statistical testing

Model performance was quantified using accuracy and class-wise precision, recall, and F1-score, with the weighted F1-score serving as the primary summary metric (27). Discriminative ability was additionally assessed using the macro-averaged one-vs-rest (OvR) area under the ROC curve (AUC). Weighted F1 was chosen as the primary model-selection metric because it reflects the imbalanced empirical class distribution and aligns with post-level classification performance; macro AUC and per-class metrics were included to evaluate minority-class discrimination and avoid relying solely on a majority-weighted summary. To characterize uncertainty in these aggregate metrics, 95% confidence intervals (CI) for weighted F1 and macro-averaged OvR AUC were computed via nonparametric bootstrapping with 2,000 resamples of the held-out test set (28).

To compare models on the same held-out test set, we conducted pairwise McNemar’s tests on per-instance correctness (29). For each model pair, a 2×2 table was formed from counts of (i) both correct, (ii) only model 1 correct, (iii) only model 2 correct, and (iv) both incorrect; McNemar’s test evaluates whether the discordant counts differ under the null of equal error rates. We used the asymptotic test with continuity correction (exact = False, correction = True; statsmodels) and applied Bonferroni adjustment to raw *p*-values across all 15 pairwise comparisons, declaring family significance at α=0.05 (equivalent unadjusted threshold: 0.05/15=0.0033). Direction of difference was interpreted from the model with higher held-out correctness and the corresponding discordant-pair balance.

### Error analysis

Because the sentiment labels are ordinal, error analysis was conducted using class-specific confusion patterns to distinguish between adjacent (e.g., negative → very negative) and distant (e.g., positive → very negative) misclassifications. This analysis aimed to identify systematic failures, such as consistent under-detection of high-distress categories, which would have important implications for potential use in monitoring workflows or supportive-care triage settings.

## Results

We first describe the distribution of sentiment labels in the four-class cancer patient and caregiver vulnerability forum corpus to contextualize class imbalance and the need for weighted evaluation. We then report held-out test-set performance for all evaluated models, including point estimates and bootstrapped 95% confidence intervals for weighted F1 and macro one-vs-rest AUC, with model selection determined exclusively on the validation set. Finally, we present statistical comparisons using Bonferroni-adjusted McNemar tests, followed by transformer-focused per-class performance and error analysis to clarify where contextual models provide meaningful gains and where residual failure modes remain.

### Distribution of sentiment labels

The finalized dataset utilized for model training and evaluation comprised a total of 10,392 instances *(four-class filtered corpus)*. Analysis of the outcome distribution revealed substantial class imbalance, consistent with the emotional profile typically observed in oncological support forums. The majority of posts were categorized as either neutral or negative, accounting for 4,375 (42.1%) and 4,112 (39.6%) instances, respectively. In contrast, the categories representing the poles of sentiment intensity were markedly less frequent: the very negative class, capturing acute psychological distress, comprised 1,155 (11.1%) instances, while the positive class was the least prevalent category with 750 (7.2%) instances. This skewed distribution underscores the necessity of employing weighted performance metrics and class-balancing strategies, as the relatively low prevalence of highly positive or highly distressed posts increases the difficulty of learning robust decision boundaries across the ordinal sentiment continuum.

### Tuned hyperparameters and overall model performance

Following the systematic optimization procedures, the optimal hyperparameter configurations for each model were identified based on validation set performance. The Logistic Regression model used a multinomial formulation with an $\ell_{2}$ penalty, a regularization strength of C=10, the saga solver, and balanced class weights. The best-performing Random Forest ensemble consisted of 100 estimators with max_depth = None, requiring a minimum of 2 samples to split an internal node and 4 samples per leaf, and employed balanced class weights to address the skewed label distribution. For LightGBM, the optimal configuration included 200 boosting iterations with a learning rate of 0.1, 31 leaves, max_depth = None, and again employed balanced class weights.

Among the transformer models, **ALBERT** achieved its best validation performance when fine-tuned with a learning rate of $1.197\times 10^{-5}$ over 4 training epochs and a dropout rate of 0.0621. **BioBERT** achieved its best validation performance with the same selected learning rate ($1.197\times 10^{-5}$), 4 training epochs, and a dropout rate of 0.0621. Finally, the custom GRU architecture attained peak performance with an embedding dimension of 158, 766 hidden units, a learning rate of $6.774\times 10^{-4}$, and 9 training epochs.

Table 2 shows that the transformer-based models achieved the strongest overall performance on the four-class sentiment task. **ALBERT** produced the highest weighted F1 (0.7667) and macro one-vs-rest AUC (0.9312), while **BioBERT** was closely comparable (weighted F1 = 0.7613; macro AUC = 0.9170) and achieved the highest accuracy/weighted recall (0.7590). These results indicate that contextual representations learned via transformer fine-tuning capture nuanced affective cues in cancer support narratives more effectively than feature-based ML baselines or the recurrent neural model, while also suggesting that biomedical-domain pretraining did not clearly exceed a compact general-purpose transformer on this label definition.

**Table 2:** Overall model performance summary on the test set with 95% bootstrap confidence intervals.

Model	Precision	Recall	Weighted F1 [95% CI]	Macro AUC [95% CI]
Logistic Regression	0.7127	0.7100	0.7109 [0.6914, 0.7304]	0.8835 [0.8711, 0.8949]
Random Forest	0.6938	0.6893	0.6853 [0.6647, 0.7057]	0.8744 [0.8626, 0.8860]
LightGBM	0.7210	0.7287	0.7226 [0.7030, 0.7420]	0.8833 [0.8712, 0.8950]
GRU	0.7046	0.7047	0.7032 [0.6837, 0.7224]	0.8555 [0.8406, 0.8693]
ALBERT	0.7875	0.7581	0.7667 [0.7487, 0.7845]	0.9312 [0.9227, 0.9391]
BioBERT	0.7665	0.7590	0.7613 [0.7431, 0.7794]	0.9170 [0.9070, 0.9271]

Among the classical baselines, **Logistic Regression** and **LightGBM** formed a comparable second tier. Logistic Regression attained a weighted F1 of 0.7109 (95% CI: 0.6914–0.7304) and a macro AUC of 0.8835 (95% CI: 0.8711–0.8949), demonstrating that TF–IDF representations coupled with a linear decision boundary provide strong discrimination in this dataset. LightGBM yielded a weighted F1 of 0.7226 (95% CI: 0.7030–0.7420) with a macro AUC of 0.8833 (95% CI: 0.8712–0.8950), alongside higher recall (0.7287 vs. 0.7100) and comparable precision (0.7210 vs. 0.7127). The overlapping confidence intervals for weighted F1 imply that the practical difference between these two approaches is modest; logistic regression offers a simpler, more interpretable baseline, while LightGBM provides a higher-capacity non-linear ensemble.

**Random Forest** and **GRU** exhibited weaker performance relative to the top-performing transformer models. Random Forest achieved a weighted F1 of 0.6853 (95% CI: 0.6647–0.7057) and macro AUC of 0.8744 (95% CI: 0.8626–0.8860). The GRU model achieved a higher weighted F1 than Random Forest (0.7032; 95% CI: 0.6837–0.7224) but the lowest macro AUC (0.8555; 95% CI: 0.8406–0.8693). Because this GRU used randomly initialized task-specific embeddings rather than pretrained word vectors, its performance should be interpreted as a lower-bound recurrent baseline rather than evidence that optimized or pretrained recurrent models cannot perform better.

### Interpretability of classical baselines via TF–IDF feature importance

Analysis of TF–IDF feature importance across the classical machine learning baselines revealed a consistent reliance on high-intensity affective and mortality-related tokens. Across logistic regression, random forest, and LightGBM, the terms *lose*, *die*, and *feel* ranked among the most influential predictors, reflecting the prominence of loss-focused and emotionally explicit narratives in cancer patient and caregiver support forums. In addition, all three models placed substantial weight on domain-relevant identifiers, including familial roles (e.g., *mom*, *mother*, *husband*) and general cancer-related terms (e.g., *cancer*, *leukemia*), suggesting that these lexical markers help contextualize caregiver and illness narratives alongside affective expression. Notably, the top-ranked features were dominated by affective and relational language rather than specific cancer subtype terminology, consistent with the supervision target: ordinal sentiment intensity is more directly signaled by emotional disclosure and caregiving context than by tumor categories, which are comparatively sparse and less discriminative for affect. This feature-level transparency provides an interpretable reference point for how sparse lexical models operationalize sentiment in this domain, with negatively valenced markers (e.g., *angry*, *sad*, *hard*) consistently associated with distress-related classifications.

The linguistic profiles surfaced by the TF–IDF baselines also align with the model failure modes observed at the sentiment extremes. Tokens such as *pass away*, *loss*, and *hurt* were prominent among high-importance features, supporting sensitivity to acute distress cues and helping explain relatively strong detection of very negative and negative posts when explicit crisis language is present. Conversely, resilience-oriented terms (e.g., *thank*, *love*, *hope*) contributed to identifying positive sentiment; however, their frequent co-occurrence with neutral informational updates and treatment narratives may reduce separability between neutral and positive categories, consistent with weaker performance on the positive class. Overall, while TF–IDF models can effectively exploit explicit lexical cues (yielding weighted F1 scores in the 0.69–0.72 range), their reliance on isolated tokens underscores the advantage of transformer-based contextual representations for capturing mixed, clause-level sentiment and multi-sentence affective signals that are common in vulnerability-oriented cancer support discourse.

### Per-class performance and statistical comparison

Pairwise McNemar tests with Bonferroni correction indicated that **ALBERT** and **BioBERT** significantly outperformed logistic regression, random forest, and GRU on the held-out test set (corrected p<0.001 for each of these transformer-vs-baseline comparisons; family-wise threshold α=0.05 across 15 comparisons). In contrast, neither transformer differed significantly from LightGBM after multiple-comparison control (ALBERT vs. LightGBM corrected p=0.1267; BioBERT vs. LightGBM corrected p=0.0785). The difference between **ALBERT and BioBERT** was also not statistically significant after correction (corrected p=1.0), suggesting that the two transformer models make broadly similar instance-level decisions on this corpus, with ALBERT exhibiting a modest but not reliably separable advantage in weighted F1 and macro AUC. Full pairwise disagreement counts, McNemar statistics, corrected p values, and directional interpretations are reported in Table 3.

**Table 3:** Pairwise McNemar tests on held-out test correctness.

Comparison	b	c	Statistic	Corrected p	Direction
LogReg vs. RF	246	203	3.929	0.7120	Not significant
LogReg vs. LightGBM	147	186	4.336	0.5596	Not significant
LogReg vs. ALBERT	198	298	19.760	$1.32\times 10^{-4}$	ALBERT > LogReg
LogReg vs. BioBERT	204	306	20.002	$1.16\times 10^{-4}$	BioBERT > LogReg
LogReg vs. GRU	308	275	1.756	1.0000	Not significant
RF vs. LightGBM	155	237	16.737	$6.44\times 10^{-4}$	LightGBM > RF
RF vs. ALBERT	186	329	39.153	$5.88\times 10^{-9}$	ALBERT > RF
RF vs. BioBERT	179	324	41.225	$2.04\times 10^{-9}$	BioBERT > RF
RF vs. GRU	291	301	0.137	1.0000	Not significant
LGBM vs. ALBERT	229	290	6.936	0.1267	Not significant
LGBM vs. BioBERT	215	278	7.797	0.0785	Not significant
LGBM vs. GRU	319	247	8.906	0.0426	LGBM > GRU
ALBERT vs. BioBERT	168	170	0.003	1.0000	Not significant
ALBERT vs. GRU	344	211	31.395	$3.16\times 10^{-7}$	ALBERT > GRU
BioBERT vs. GRU	338	203	33.190	$1.25\times 10^{-7}$	BioBERT > GRU

LogReg, logistic regression; RF, random forest; LGBM, LightGBM.

b is the number of test instances correctly classified only by the first-listed model, and c is the number correctly classified only by the second-listed model. Statistics use McNemar’s test with continuity correction. Corrected p values are Bonferroni-adjusted across 15 pairwise comparisons; the family-wise significance threshold is α=0.05.

ALBERT and BioBERT achieved closely comparable performance for four-class sentiment classification in the cancer patient and caregiver vulnerability forum dataset. ALBERT was modestly higher on weighted F1 (0.7667 vs. 0.7613) and macro AUC (0.9312 vs. 0.9170), whereas BioBERT was slightly higher on accuracy/weighted recall (0.7590 vs. 0.7581). This pattern supports that transformer fine-tuning is beneficial for this task but that the incremental advantage of biomedical-domain pretraining over a compact general transformer is limited in this particular dataset.

Table 4 summarizes class-specific precision, recall, F1, and support for ALBERT and BioBERT. For ALBERT, negative (class 1) and neutral (class 2) posts achieved F1-scores of 0.7659 and 0.8181, respectively. For very negative content (class 0), ALBERT showed high recall (0.7736) with precision of 0.7130, while BioBERT showed slightly higher recall for this class (0.8019) with precision of 0.6589. Performance remained weakest for positive posts (class 3), although ALBERT achieved higher positive-class recall than BioBERT (0.7517 vs. 0.5379), indicating that the least prevalent class remained difficult to separate and that model choice changes the balance of missed positive posts vs. false positives.

**Table 4:** Class-specific performance on the test set (ALBERT & BioBERT).

Class	Precision	Recall	F1-score	Support
Panel A. ALBERT				
Very Negative	0.7130	0.7736	0.7421	212
Negative	0.8253	0.7144	0.7659	886
Neutral	0.8354	0.8014	0.8181	836
Positive	0.3893	0.7517	0.5129	145
Panel B. BioBERT				
Very Negative	0.6589	0.8019	0.7234	212
Negative	0.7882	0.7393	0.7630	886
Neutral	0.8252	0.8074	0.8162	836
Positive	0.4535	0.5379	0.4921	145

**Figure 1:**
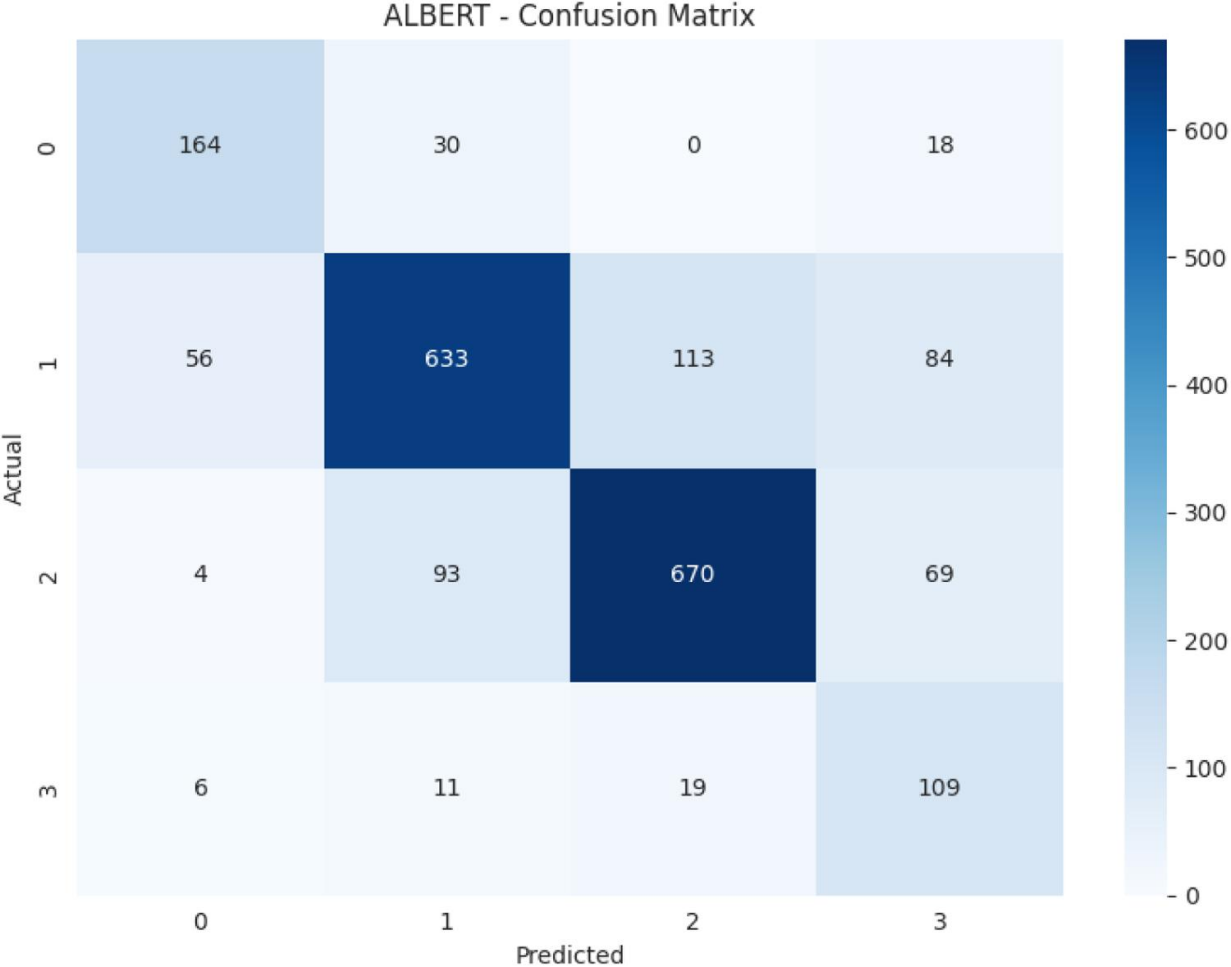
Confusion matrix for ALBERT on the held-out test set. Sentiment labels are encoded as ordinal classes: 0 = very negative, 1 = negative, 2 = neutral, and 3 = positive.

**Figure 2:**
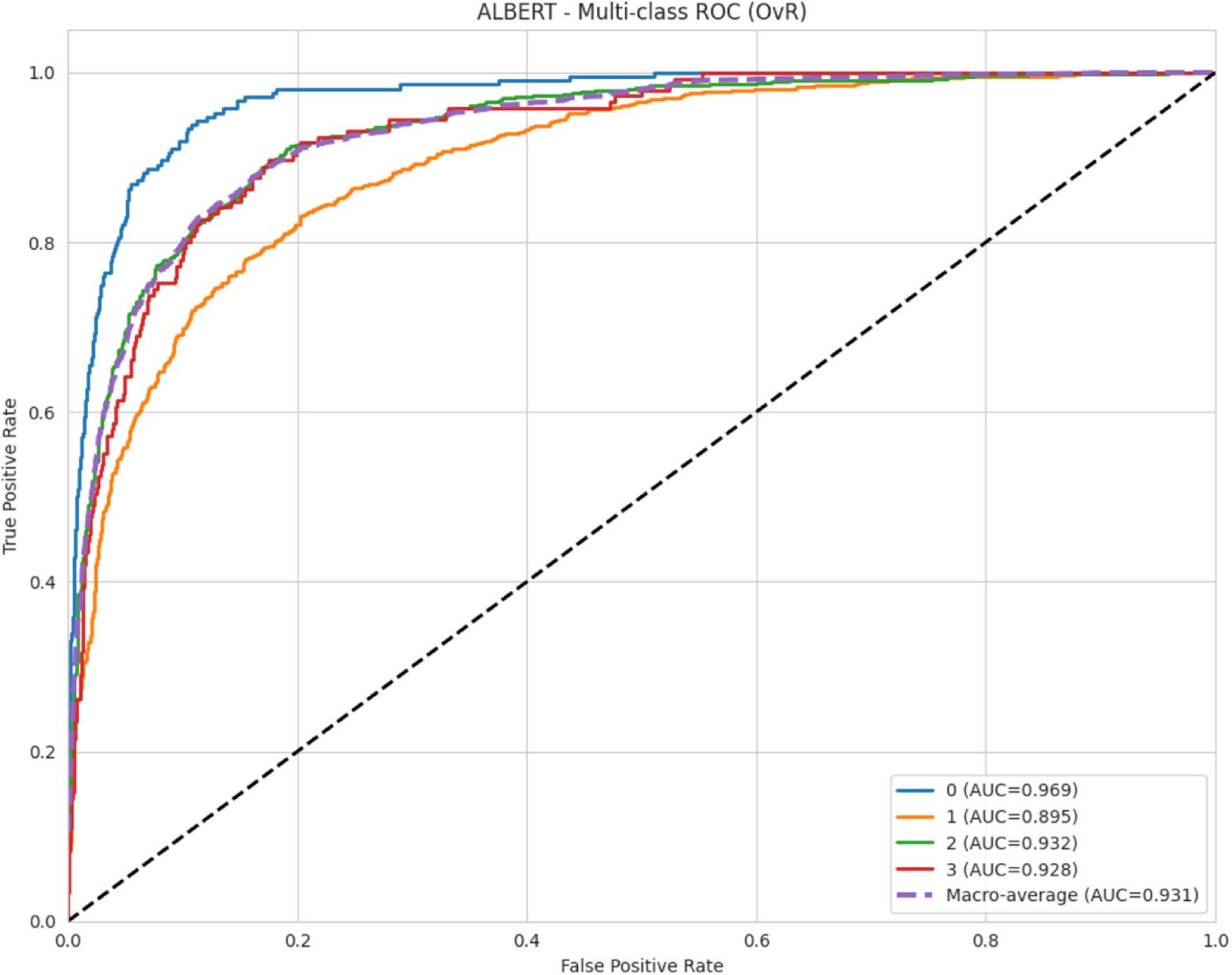
One-vs-rest ROC curves for ALBERT on the held-out test set. Sentiment labels are encoded as ordinal classes: 0 = very negative, 1 = negative, 2 = neutral, and 3 = positive.

**Figure 3:**
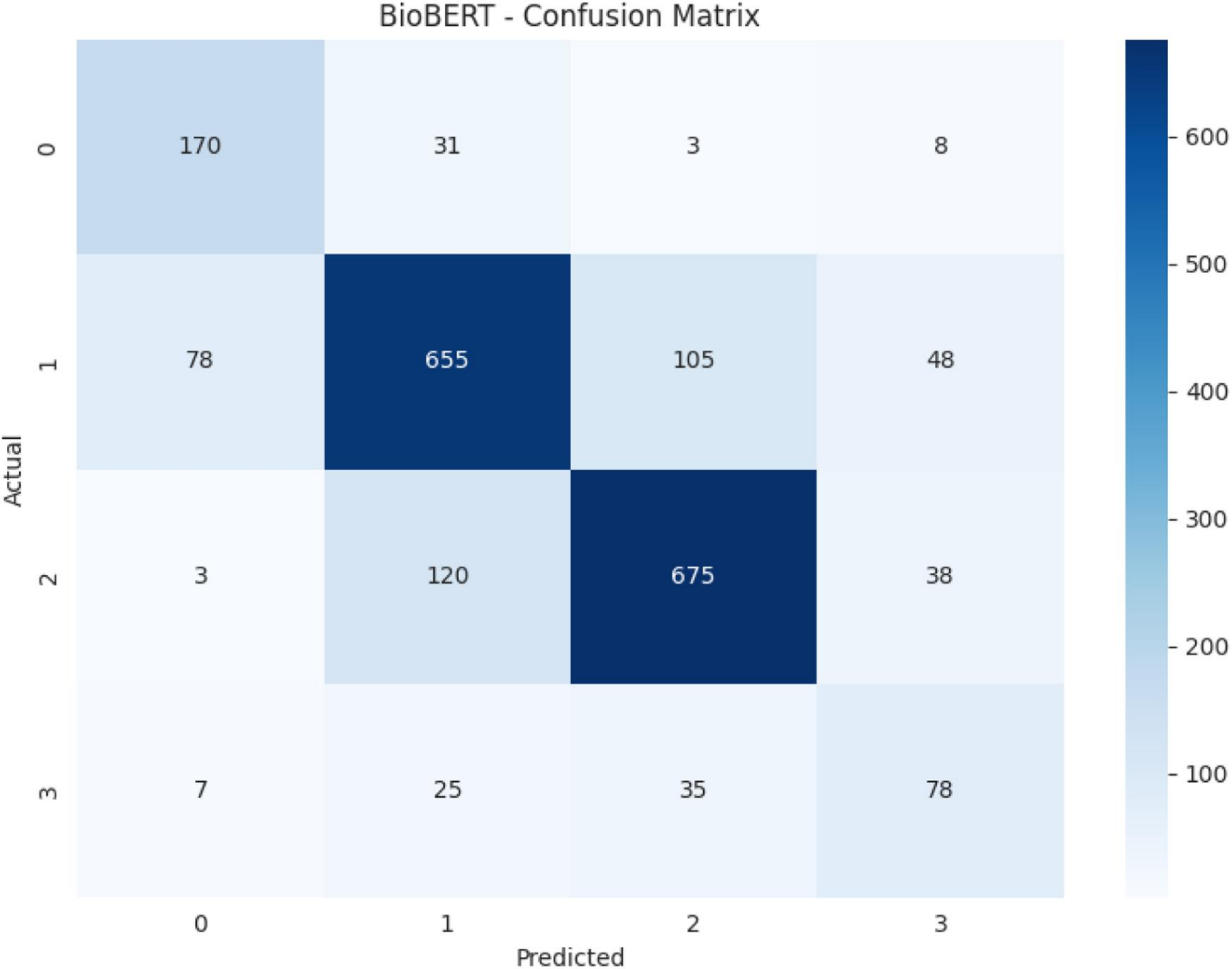
Confusion matrix for BioBERT on the held-out test set. Sentiment labels are encoded as ordinal classes: 0 = very negative, 1 = negative, 2 = neutral, and 3 = positive.

**Figure 4:**
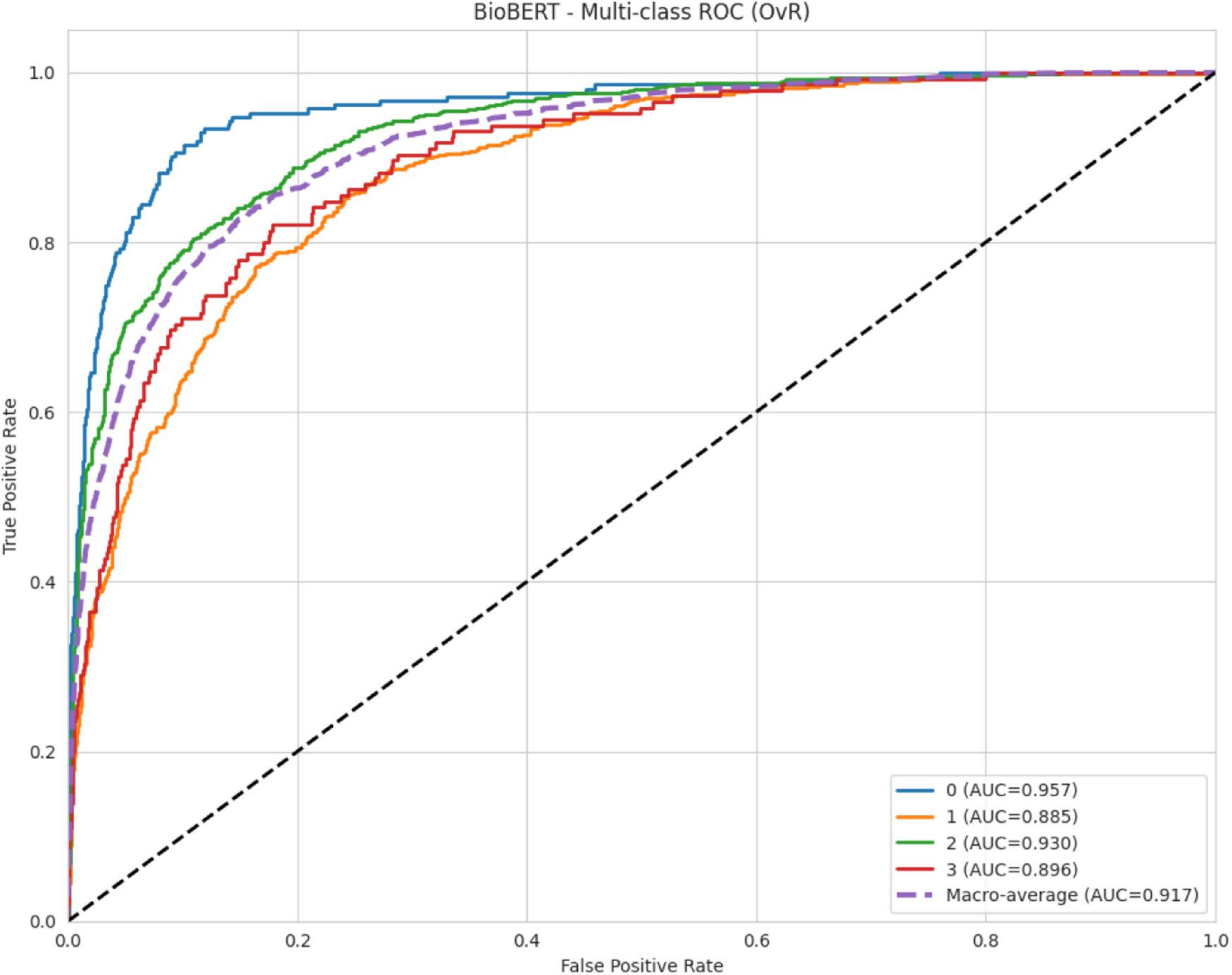
One-vs-rest ROC curves for BioBERT on the held-out test set. Sentiment labels are encoded as ordinal classes: 0 = very negative, 1 = negative, 2 = neutral, and 3 = positive.

Both panels use the same held-out test set. The support column gives the ground-truth class distribution in the unified split (very negative = 212, negative = 886, neutral = 836, positive = 145). Same-split ALBERT and BioBERT visual diagnostics are provided in Figures 1–4.

Figures 1–4 provide complementary same-split visual diagnostics for ALBERT and BioBERT. The confusion matrices show that many misclassifications occur between adjacent sentiment categories, with prominent confusion at the Neutral → Negative and Negative → Very negative boundaries. The one-vs-rest ROC curves are consistent with the strong macro AUC values reported in Table 2, indicating good overall separability across classes, with comparatively weaker discriminability for the positive class.

### Error analysis

To understand *where* transformer fine-tuning improves over non-contextual baselines and *where* residual weaknesses remain, we interpreted errors through the ordinal structure of the four-class sentiment scale. Consistent with the McNemar results, **ALBERT** produced more correct predictions than the TF–IDF baselines and the GRU, indicating that contextual representations capture discourse-level cues (e.g., multi-sentence affect, implicit distress, mixed coping language) that are not well represented by sparse lexical features or a shallow recurrent encoder. **BioBERT** showed similar qualitative behavior, reinforcing that contextual modeling is the primary driver of performance gains in this setting.

Despite these advantages, transformer errors were not random; they concentrated around boundary regions between *adjacent* sentiment categories. For ALBERT, 380 of 503 errors (75.5%) were adjacent on the ordinal scale and 123 (24.5%) were non-adjacent. For BioBERT, 407 of 501 errors (81.2%) were adjacent and 94 (18.8%) were non-adjacent. The dominant adjacent misclassifications involved Neutral→Negative and Negative→Very negative transitions, indicating a systematic tendency for some mid-spectrum narratives to shift toward higher distress intensity.

Both transformers also remained comparatively weaker on the positive category. ALBERT correctly identified 109 of 145 positive posts and BioBERT correctly identified 78 of 145, leaving 36 and 67 positive-post errors, respectively. Among ALBERT’s positive-class errors, 19 were Positive → Neutral, 11 were Positive → Negative, and 6 were Positive → Very negative; for BioBERT, the corresponding counts were 35, 25, and 7.0 Thus, although Positive → Neutral errors are expected given semantic proximity, Positive → Negative and Positive → Very negative errors are more consequential because they reverse the apparent affective direction of a post. Collectively, the error structure suggests that further refinement should prioritize decision-boundary improvements, particularly around 2↔1 and 1↔0, while also examining non-adjacent positive-to-negative errors as operationally important failure modes.

#### Illustrative all-model failure cases

We additionally inspected representative test instances for which all evaluated architectures (logistic regression, random forest, LightGBM, GRU, ALBERT, and BioBERT) produced incorrect predictions. These examples were used qualitatively to illustrate semantic patterns that remained difficult even for transformer-based encoders.

**Case 1: Medical keyword masking (ID 77).** *Text (excerpt):* “just want to let everyone know that I finally get a doc… he was really concerned with my elevated white blood count and my low reb blood count… he really listen…” *True label:* Positive (relief/coping). *Predicted label:* Negative (all models). This case illustrates a *medical polarity* failure mode: high-salience clinical abnormality terms (e.g., *elevated white blood count*, *low red blood count*) dominate the lexical and contextual signal, leading models to prioritize biomedical crisis cues over affective relief toward receiving attentive care. The result is a systematic misreading in which clinical terminology overwhelms coping-oriented sentiment.

**Case 2: Boundary ambiguity in first-post disclosure (ID 13).** *Text (excerpt):* “hi there I am new here 34 year old male… for the past two month I have been have various…” *True label:* Neutral (introduction/information seeking). *Predicted label:* Negative (all models). This case reflects an intrinsic boundary ambiguity common in support forums: first-post narratives often contain neutral biographical framing and information-seeking intent, yet necessarily include symptom descriptions and medical uncertainty. Models consistently interpret symptom disclosure as affective negativity, suggesting that “neutral” in cancer-related discourse can be semantically entangled with concern and uncertainty even when emotional tone is not explicitly negative.

## Discussion

This study evaluated representative machine learning and deep learning approaches for four-class ordinal sentiment classification in a cancer patient and caregiver vulnerability forum dataset using a holdout protocol, uncertainty estimation, and paired hypothesis testing. Overall, transformer fine-tuning yielded the strongest performance, with **ALBERT** producing the highest aggregate performance and **BioBERT** following closely. This pattern is consistent with the view that vulnerability-related affect in oncology support forums is driven by both general contextual sentiment cues and domain-specific medical language. Because all performance estimates come from a single randomized split, model ranking should be interpreted as controlled held-out benchmark evidence rather than a resampling-stable ordering.

Pairwise McNemar tests (Bonferroni-corrected) indicated that **ALBERT** and **BioBERT** significantly outperformed logistic regression, random forest, and GRU in per-instance correctness, supporting that their improvements over these comparators are unlikely to be attributable to chance variation. However, neither transformer differed significantly from LightGBM after multiple-comparison adjustment, and the difference between **ALBERT and BioBERT was not statistically significant**. In practical terms, this suggests that the incremental value of biomedical pretraining over a compact general-purpose transformer is modest for this dataset and label definition. Recent mental-health NLP benchmarking similarly cautions that model ranking can depend on preprocessing, class imbalance, and evaluation protocol rather than domain pretraining alone (19, 20). One plausible explanation is that many decision-relevant cues in support forums are affective, relational, and discourse-level rather than strictly biomedical. ALBERT’s parameter-efficient general language representations may therefore capture expressions of fear, relief, gratitude, and uncertainty sufficiently well (17), while BioBERT’s biomedical-domain pretraining may help with clinical terminology but not necessarily with mixed-valence peer-support narratives (18). Together, these findings reinforce the value of contextual encoding for oncology support narratives, where meaning is often distributed across clauses and sentences and where posts commonly blend informational content with emotionally salient disclosures.

### Practical implications for vulnerability-oriented monitoring

From an operational perspective, one consequential finding is the strong performance of the transformer models on the supportively salient high-distress category. BioBERT showed slightly higher recall for *very negative* posts, whereas ALBERT achieved higher precision for this class and the strongest aggregate weighted F1 and macro AUC. This indicates that fine-tuned transformers can support screening applications in which the primary goal is to surface potentially high-need patient or caregiver narratives for timely review. In real-world workflows, such outputs could be used to prioritize moderator attention, route posts to supportive resources, or trigger follow-up by trained staff within an online community. Importantly, the confusion patterns indicate that most errors remain adjacent on the ordinal scale, reducing the likelihood of large-distance misclassifications (e.g., very negative → positive) that would be most harmful for triage.

However, sensitivity introduces an operational trade-off. BioBERT’s slightly higher recall for very negative posts was accompanied by lower precision for this class, indicating a greater tendency to over-flag some non-very-negative posts as very negative. ALBERT, by contrast, showed a somewhat more balanced profile for very negative content and stronger positive-class recall, which may be useful when both high-distress detection and preservation of resilience-oriented content matter. These complementary behaviors suggest that model selection should be driven by workflow priorities: BioBERT may be useful when sensitivity to very negative posts is prioritized, whereas ALBERT may be preferable when aggregate discrimination and positive-class recovery are also important. In all cases, these models are best positioned as front-line *decision-support* components rather than autonomous decision systems, and deployment should incorporate calibrated operating points and human oversight.

### Residual weaknesses and error-informed refinement

Although the transformer models performed best overall, their remaining failure modes were systematic and informative. Errors concentrated at adjacent decision boundaries, notably Neutral ↔ Negative and Negative ↔ Very negative. This boundary ambiguity is expected in vulnerability-related discourse: posts frequently blend factual updates (treatment, symptoms, prognosis discussions) with uncertainty, fear, and coping language, making sharp categorical boundaries intrinsically difficult. When vulnerability cues (e.g., fatigue, fear, side effects) co-occur with neutral informational content, the Neutral/Negative and Negative/Very negative boundaries become ambiguous even for contextual encoders.

These patterns point to actionable refinements. Probability calibration and class-aware decision policies may help reduce over-escalation without sacrificing sensitivity to acute distress, and ordinal learning objectives may further discourage large-distance mistakes while preserving fine-grained ranking. In addition, targeted annotation of borderline examples near the 0/1 and 1/2 boundaries may yield disproportionate gains by clarifying labeling rules for mixed-content posts and improving consistency in the supervision signal.

Distinguishing *positive* from *neutral* remained challenging, but the error analysis also showed a smaller set of positive-to-negative errors. Positive → Neutral errors are consistent with cancer forum language where gratitude, hope, and coping often co-occur with pragmatic updates and ongoing concerns. Positive → Negative errors are more consequential because they can invert the affective interpretation of a post. The illustrative medical-keyword case shows one plausible mechanism: a post expressing relief about finally receiving attentive care may also contain abnormal laboratory values or symptom descriptions, causing models to over-weight biomedical risk cues and under-weight relief or coping language. If identifying resilience-oriented content is a primary objective (e.g., to support peer reinforcement or to study protective factors), future work should explicitly target this boundary through refined labeling guidance, targeted enrichment of positive examples, and potentially auxiliary objectives that help separate affective tone from informational content.

Although BioBERT incorporates domain-adapted pretraining, its performance was not statistically distinguishable from ALBERT under Bonferroni-adjusted McNemar testing. Practically, this suggests that model selection should be guided less by small differences in aggregate metrics and more by deployment constraints and workflow priorities. The executed notebook provides preliminary deployment indicators from the held-out prediction workflow: batched prediction over 2,079 test posts took approximately 28 s for ALBERT (batch size 32; about 13.5 ms/post), 25 s for BioBERT (batch size 32; about 12.0 ms/post), and 2 s for the GRU (batch size 16; about 1.0 ms/post). The serialized model artifacts also differed substantially in size: 46.8 MB for ALBERT, 433.3 MB for BioBERT, and 14.8 MB for the GRU, compared with 0.3 MB for logistic regression, 7.2 MB for random forest, and 3.1 MB for LightGBM. For sparse baselines, these file sizes refer to the saved classifier objects and do not include a complete text-preprocessing service. These notebook-derived values are approximate because they reflect the experimental prediction workflow rather than a production serving benchmark and do not measure peak runtime memory. Even so, they support a practical distinction: when storage, maintainability, or deployment footprint are limiting factors, ALBERT offers comparable accuracy to BioBERT with a much smaller serialized artifact, whereas BioBERT may still be attractive when very-negative sensitivity or richer biomedical context is prioritized. Hardware-specific latency, throughput, and memory profiling should therefore be performed before operational use.

The observed error modes motivate safeguards for real-world integration. First, high sensitivity for the *very negative* class supports screening, but predictions should not be interpreted as clinical diagnosis; escalation policies should be calibrated to the capacity and goals of the reviewing team. Second, all-model failure cases (e.g., medical keyword masking and first-post boundary ambiguity) indicate that certain posts remain challenging even for transformers, reinforcing a human-in-the-loop design. A practical policy is to route posts with low predicted confidence or medically salient abnormality cues to manual review, regardless of the predicted label, to reduce the risk of misinterpreting coping-oriented messages as distress or vice versa. Together, these safeguards align model outputs with decision-support use rather than autonomous intervention.

### Limitations

This study has several limitations. First, the dataset is sourced from a public corpus, and the extent to which its posts represent broader cancer populations, platforms, and cultural contexts is uncertain; external validity should therefore be established through evaluation on additional online communities. Public social-data corpora can reflect platform-specific participation patterns, moderation practices, and demographic biases that are not visible from text labels alone (30, 31). Second, the present study inherits labels from the source dataset and does not independently audit annotation reliability, adjudicate disagreements, or validate the labels against clinical assessments. The source dataset record also does not report inter-annotator agreement statistics for the two annotators, so the extent to which label noise constrains model performance remains unknown. Sentiment labels therefore provide a proxy for vulnerability intensity but do not constitute clinical diagnosis or definitive measures of psychosocial distress. Third, the unit of analysis is the post rather than the user; because user identifiers were not available for grouped splitting, possible dependence among posts from the same participant cannot be excluded. Fourth, class imbalance, particularly the low prevalence of positive posts, likely constrained minority-class performance and may bias models toward mid-spectrum predictions. We used class weighting but did not evaluate oversampling, synthetic minority examples, or back-translation augmentation, because those strategies can change the semantic distribution of short forum narratives and require separate validation. Fifth, evaluation was conducted using a single randomized holdout split; while bootstrapping characterizes uncertainty on the fixed test set, repeated resampling, grouped user-level splitting where identifiers are available, and external validation would strengthen claims of robustness and generalizability. Sixth, although the notebook provides approximate batched prediction times and serialized artifact sizes, the study did not conduct dedicated production profiling of peak runtime memory, cold-start behavior, throughput under concurrent load, or hardware-specific serving latency, which are necessary for choosing among models in deployable screening workflows. Finally, the analysis does not address temporal drift: forum language and community norms can evolve, and model stability under such shifts remains untested.

### Future directions

Several extensions would improve future translation and scientific understanding. (i) **Calibration and operating-point tuning:** assess probability calibration and tune decision thresholds for specific workflow constraints (e.g., top-k review, high-recall triage, or balanced precision–recall), explicitly reflecting the relative costs of false positives vs. false negatives. (ii) **Ordinal and cost-sensitive learning:** incorporate ordinal losses or cost-sensitive objectives aligned with the salience of high-distress detection to reduce systematic boundary over-escalation. (iii) **Data and label refinement:** enrich minority classes (especially positive), evaluate augmentation or oversampling strategies with semantic-quality checks, and improve labeling guidance for boundary cases; targeted annotation of difficult posts near the 0/1 and 1/2 boundaries may yield disproportionate gains. (iv) **Generalization and robustness:** validate the transformer models across repeated random splits, platforms, cancer contexts, and time periods, and evaluate performance under domain shift and temporal drift. (v) **Deployment profiling and human-in-the-loop evaluation:** extend the preliminary notebook timing and artifact-size checks with hardware-specific profiling of inference latency, runtime memory, throughput, reviewer workload, timeliness of response, and downstream supportive outcomes when model outputs are integrated into moderation or supportive care workflows, with safeguards for privacy, consent, and responsible use. Future extensions could also integrate lightweight structured context (e.g., patient vs. caregiver role) alongside text to further reduce ambiguity at adjacent decision boundaries.

In conclusion, transformer fine-tuning improved held-out performance for ordinal sentiment classification in cancer patient and caregiver vulnerability forum narratives under a single 60/20/20 split, with ALBERT and BioBERT showing statistically significant gains over logistic regression, random forest, and GRU, but not over LightGBM after Bonferroni correction. ALBERT delivered the strongest weighted F1 and macro AUC, while BioBERT remained closely comparable and showed slightly higher recall for very negative posts. These characteristics support the use of transformers as screening components for vulnerability-oriented monitoring, while emphasizing the importance of calibration, boundary-focused refinement, robustness testing, deployment profiling, and human oversight before real-world deployment.

## Data Availability

The original contributions presented in the study are included in the article/Supplementary Material, further inquiries can be directed to the corresponding author.
